# Brain tumor segmentation by combining MultiEncoder UNet with wavelet fusion

**DOI:** 10.1002/acm2.14527

**Published:** 2024-09-16

**Authors:** Yuheng Pan, Haohan Yong, Weijia Lu, Guoyan Li, Jia Cong

**Affiliations:** ^1^ Computer and Information Engineering Department Tianjin Chengjian University Tianjin China

**Keywords:** 3D discrete wavelet transformer, brain tumor segmentation, multi‐encoder

## Abstract

**Background and objective:**

Accurate segmentation of brain tumors from multimodal magnetic resonance imaging (MRI) holds significant importance in clinical diagnosis and surgical intervention, while current deep learning methods cope with situations of multimodal MRI by an early fusion strategy that implicitly assumes that the modal relationships are linear, which tends to ignore the complementary information between modalities, negatively impacting the model's performance. Meanwhile, long‐range relationships between voxels cannot be captured due to the localized character of the convolution procedure.

**Method:**

Aiming at this problem, we propose a multimodal segmentation network based on a late fusion strategy that employs multiple encoders and a decoder for the segmentation of brain tumors. Each encoder is specialized for processing distinct modalities. Notably, our framework includes a feature fusion module based on a 3D discrete wavelet transform aimed at extracting complementary features among the encoders. Additionally, a 3D global context‐aware module was introduced to capture the long‐range dependencies of tumor voxels at a high level of features. The decoder combines fused and global features to enhance the network's segmentation performance.

**Result:**

Our proposed model is experimented on the publicly available BraTS2018 and BraTS2021 datasets. The experimental results show competitiveness with state‐of‐the‐art methods.

**Conclusion:**

The results demonstrate that our approach applies a novel concept for multimodal fusion within deep neural networks and delivers more accurate and promising brain tumor segmentation, with the potential to assist physicians in diagnosis.

## 1 INTRODUCTION

**FIGURE 1:**
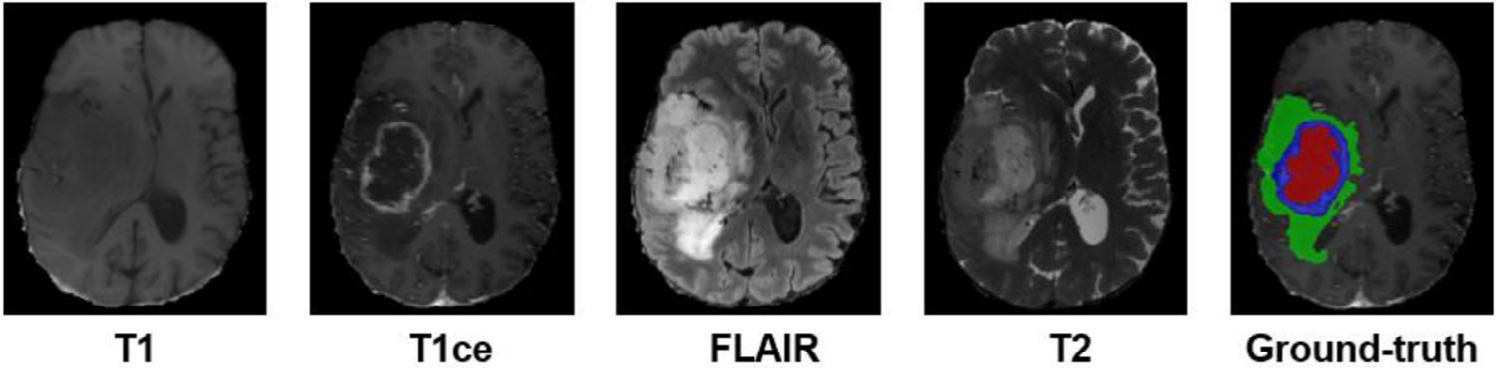
A sample from the BraTS2021 training dataset is depicted, exhibiting images arranged in the following order: T1, T1ce, T2, FLAIR, and the Ground Truth, which embodies the doctor's manual segmentation annotation. The color codes red, blue, and green correspond to the representation of necrotic tissue, enhancing tumor and edema, respectively.

Brain tumors, notably gliomas, represent a significant menace to human health, marked by their status as the most prevalent primary malignant intracranial tumors associated with elevated rates of both mortality and morbidity.^1^ Among gliomas, grades I and II are classified as low‐grade gliomas (LGGs), and grades III and IV are high‐grade gliomas (HGGs). The best treatment depends on the early diagnosis of gliomas and their grading.^2^ Within diagnostic practices, magnetic resonance imaging (MRI) is a widely utilized method for detecting brain tumors. The conventional MRI imaging protocol for brain analysis encompasses a series of sequences, including T1‐weighted (T1), T1‐weighted contrast‐enhanced (T1ce), T2‐weighted (T2), and T2 fluid‐attenuated inversion recovery (FLAIR),^3^ different modalities of images capture different pathological information and complement each other effectively. For example, T2 and FLAIR are suitable for detecting tumors with peritumoral edema, whereas T1 and T1c effectively identify tumor cores (TCs) without peritumoral edema. As shown in Figure 1, traditional brain tumor segmentation necessitates the manual segmentation of the pathological area by experts, performed slice by slice, for diagnosis, risk evaluation, and treatment strategizing.^4^ Manually segmenting tumors is complex and time‐consuming and depends on the expertise of professionals.^5^ In contrast, automated segmentation can assist physicians in quickly detecting and accurately locating tumors, improving diagnostic accuracy and efficiency, and thus better assisting in developing surgical and radiotherapy plans and improving patient survival rates. As a result, the automation of tumor segmentation holds significant importance in medical imaging applications.^6^, ^7^

Recently, advancements in medical image segmentation have been made through deep learning methodologies, notably exemplified by convolutional neural networks (CNNs) and Transformer architectures. The UNet^8^ model stands as a prominent instance of a CNN that adeptly balances global and local information through interconnecting lower‐level and higher‐level features using skip connections, and its variations^9^, ^10^, ^11^ have demonstrated remarkable segmentation performance for brain tumors. Tseng et al.^12^ integrated 2D CNN with long short‐term memory (LSTM) to harness multimodal data and sequential information extracted from contiguous image slices. Despite this approach, the methods based on 2D networks remain insufficient in comprehensively incorporating the spatial semantic information inherent in 3D MRI datasets. In a separate study, Çiçek et al.^13^ employed 3D convolution and pooling operations for acquiring richer spatial semantic information, thereby enhancing the contextual awareness within the dataset. The attention‐based transformer model can efficiently capture long‐range dependencies between voxels in medical image datasets. Yet, owing to their elevated complexity and the absence of inductive bias properties, transformers might not achieve performance comparable to CNNs, particularly in medical datasets containing relatively limited labeled samples. Some approaches^14^, ^15^, ^16^ combine CNNs and transformers to reduce computational overhead and prevent overfitting while taking advantage of convolutions' inductive bias properties and transformers' self‐attentive long‐range dependencies.

Thus far, the segmentation of brain tumors has proven to be a complex undertaking due to various inherent challenges. Primarily, many methods^17^, ^18^, ^19^ rely on an early fusion approach, which involves merging the four MRI sequences (T1, T2, T1ce, and FLAIR) into the network model after fusion at the channel layer. This approach implicitly assumes that the different modal relationships are simply linear. However, the different image acquisition processes make the relationships between different modalities more complex than linear. As Srivastava and Salakhutdinov^20^ mentioned in multimodal scenarios, it is difficult to tap into the nonlinear relationships between low‐level features of different modalities by fusing them at an early stage. Therefore, fusing high‐level features at a later stage can better explain the complex relationships between different modalities.^21^ Besides, using neural networks for modal fusion involves many convolutional layers and often produces redundant features. On the other hand, wavelet transform‐based fusion methods can extract intrinsic multimodal features from the transform domain, leading to fusion results that are resistant to interference.^22^ As a result, we integrated the wavelet transform module into the neural network at the modal fusion stage.

Second, it involves the delineation of multiple subregions, encompassing the TC and edema, collectively constituting the entirety of the whole tumor (WT). The TC comprises both enhancing tumor (ET) and necrotic regions, adding a layer of complexity to the segmentation process. Moreover, the task is compounded by indistinct boundaries, variable sizes, and diverse spatial distributions of the tumor, necessitating precise voxel segmentation. Achieving this precision requires accounting for the comprehensive global or long‐range dependencies among the voxels within the tumor and its respective subregions.

In response to the above observations, this article proposes a multi‐modal fusion segmentation network with multiple encoders and a single decoder; each encoder corresponds to capturing modality‐specific features. The core of this network architecture is a 3D wavelet fusion module (WFM) based on a late fusion strategy, which is responsible for fusing image features at different levels for each encoder and then connecting the fused feature representation to the decoder. Simultaneously, a simplified yet efficient 3D self‐attention module is incorporated to capture long‐range dependencies among tumor voxels efficiently, enhancing the network's capacity to access comprehensive global semantic features. The primary contributions of our research are outlined as follows:

(1) An encoder structural model with multiple encoders is presented to capture the distinct features of various modalities. A novel 3D WFM is then designed to integrate the semantic features of various modalities by connecting the fused features to the decoder and fusing features between multiple encoders, based on the late fusion strategy.

(2) A 3D Global Context‐Aware Module (GCAM) is introduced to capture the extensive inter‐voxel long‐range dependencies within the model at high‐level features of the modal state.

(3) Executing experiments on the BraTs2018 and BraTS2021 datasets, showcasing competitive segmentation accuracy. Our model achieves Dice scores for ET, WT, and TC that surpass those obtained by mainstream networks.

## 2 RELATED WORK

### 2.1 Fusion strategy of multimodal feature

**FIGURE 2:**
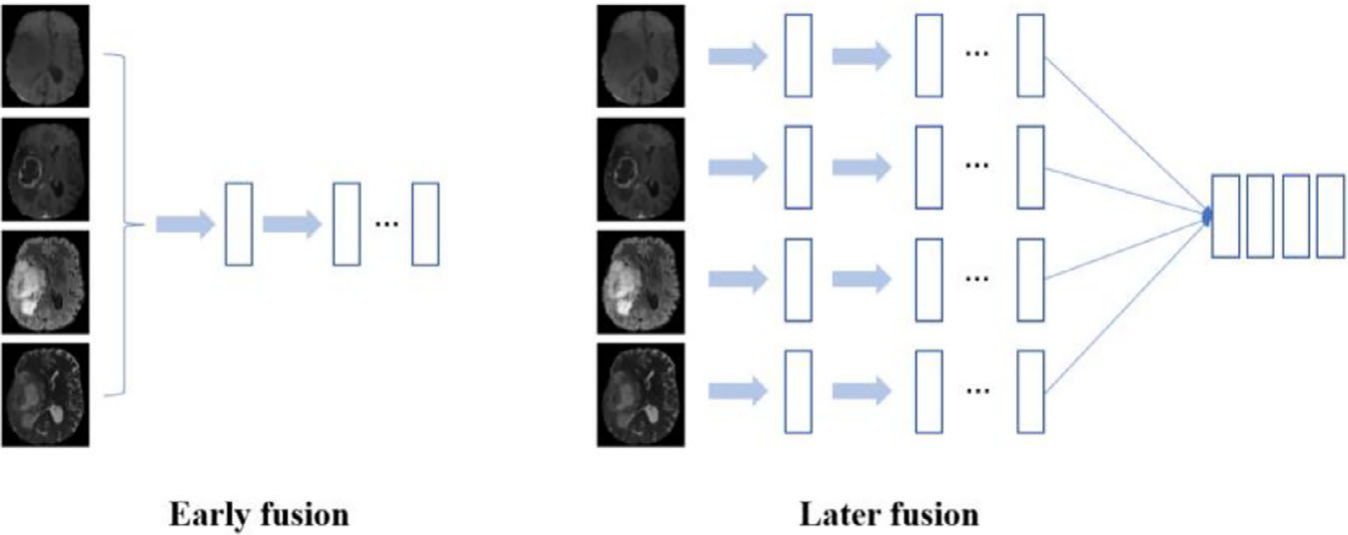
Early fusion and late fusion (early fusion is the combined input of multiple modalities into a network, and late fusion is the separate input of multiple modalities into networks).

The fusion of multimodal information is an imperative requirement for precise disease diagnosis and radiation therapy^23^ and can be categorized into early fusion strategies and late fusion strategies (see Figure 2). The later stages are more focused on integrating modalities than the early integration strategies, NestedFormer^24^ employs a nested network approach incorporating a cross‐modal attention mechanism to compute global interrelationships among diverse modalities. Similarly, Li et al.^25^ introduced a unimodal network to discern the associations between the characteristics of individual modalities and tumor subregions during multimodal fusion. This article embeds the 3D wavelet transform into the neural network as a modal fusion scheme based on the late fusion strategy. The three‐dimensional discrete wavelet transform (3D‐DWT) extends the principles of the 2D‐DWT to process volumetric data. The pseudo‐codes for implementing the 3D‐DWT are depicted in Table 1.

**TABLE 1:** Pseudo‐code for 3D‐DWT.

Algorithm 1: Pseudo‐codes for 3D‐DWT	
1:	Input the three‐dimensional data D = [D_x_, D_y_, D_z_];
2:	for z = 1 to D_z_:
3:	D(:,:, z) = 2D‐DWT(D(:,:, z));
4:	End
5:	for x = 1 to D_x_:
6:	for y = 1 to D_y_:
7:	D(x, y,:) = 1D‐DWT(D(x, y,:));
8:	End
9:	End
10:	Output the 3D‐DWT coefficients;

### 2.2 Wavelet transform in neural network

Wavelet transform^26^ has been widely utilized in neural networks due to its excellent mathematical background. Researchers have combined these neural network models with the wavelet transform method to achieve promising outcomes.^27^, ^28^, ^29^ Liu et al.^30^ embedded wavelets into CNNs for image denoising. Hu et al.^31^ utilized wavelet multi‐scale feature information for the segmentation of COVID‐19. To address the problem of boundary blurring due to low contrast of pancreatic images, a multi‐input module based on wavelet decomposition is designed so that the network pays more attention to high‐frequency texture information.^32^ Some methods^33^, ^34^ combine spatial and transform domain features to extract features from the discrete wavelet transform (DWT) while preserving the spatial representation obtained from the convolution operation, and the effectiveness of this method has been verified in medical datasets. In the field of 2D image fusion, Ding et al.^27^ extracted high and low‐frequency information using wavelet transform for deep modal features for feature fusion.

### 2.3 Self‐attention mechanism

Combining local and global features is essential in dense prediction tasks,^35^ particularly in 3D medical image segmentation. Convolutional kernels have limited receptive fields and thus require many layers to connect regions across the image, which results in poor results.^36^ Some investigations have employed pure transformer architectures founded on self‐attention, exemplified by the Swin Transformer.^37^ However, CNN with inductive bias properties performs better on small medical image datasets than transformers. Hatamizadeh et al.^15^ proposed a hybrid architecture combining the advantages of local convolution and global attention. Ismayl et al.^38^ employed the Fast Fourier Transform instead of multi‐head attention to capture global dependencies, and a convolutional network to obtain local information. Similarly, Wang et al.^16^ utilized 3D CNNs to extract local features encompassing space and depth, integrating low‐resolution high‐level features to more accurately represent medical images. These features are channeled into a transformer to model global features, and a decoder partly upsamples the output to get segmentation results. We are inspired by Gcnet^39^ to introduce a simplified global attention module for modeling long‐range dependencies.

## 3 METHOD

### 3.1 The proposed network architecture

**FIGURE 3:**
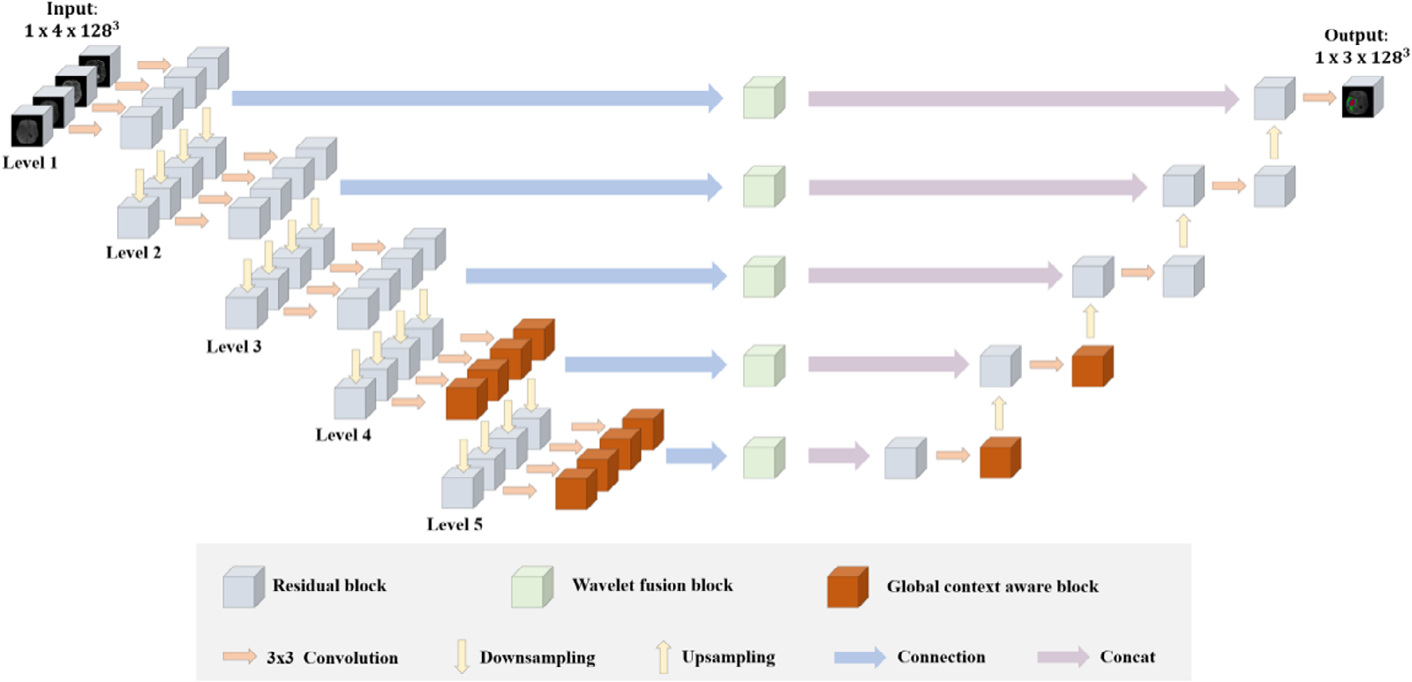
Overview of our proposed architecture. The whole network has five levels. In the first three layers, only residual blocks (grey) extract features, and in the last two layers, a global context‐aware module (orange) captures global information. The wavelet fusion module (green) fuses the modal features extracted by multiple encoders and concat the fused features to the decoder.

Figure 3 demonstrates the brain tumor segmentation model that utilizes a multi‐encoder single‐decoder structure. This model consists of five levels, with the low level (level 1 to level 3) mainly exhibiting shallow features of the tumor, such as its edges and texture. At this level, the four modal data are inputted into four encoders, and residual blocks (gray, conv+normalization+relu) and downsampling are applied to extract features. Furthermore, the high level (level 4 to level 5) provides advanced semantic features that contain global information. The Global context‐aware module (orange) is utilized at a high level to extract information and avoid a heavier computational burden. Furthermore, a WFM (depicted in green) combines the features extracted by the encoders, integrating the fused features into the decoder for further segments.

### 3.2 3D wavelet fusion module

**FIGURE 4:**
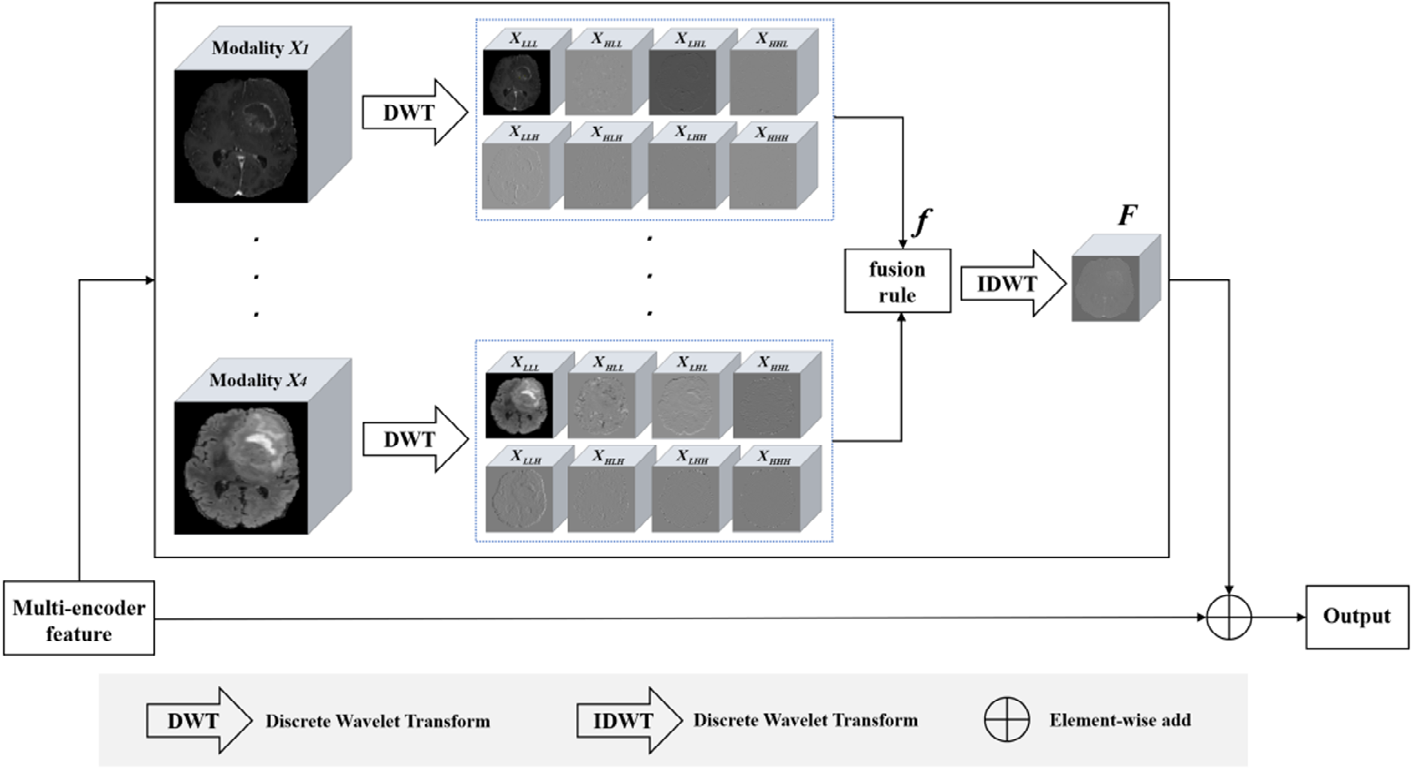
The 3D wavelet fusion module.

The 3D WFM is shown as Figure 4. At each level, four modal features ($X_{i},i\in \left\{1,2,3,4\right\}$) are obtained by 3D DWT (3D‐DWT) with eight subbands, including one low‐frequency $X_{LLL}$ and seven high‐frequency $X_{HLL}$, $X_{LHL}$, $X_{HHL}$, $X_{LLH}$, $X_{HLH}$, $X_{LHH}$, $X_{HHH}$. After the fusion rule f, the fusion feature F is obtained by the inverse discrete wavelet transform (IDWT). For the fusion rule f, the low‐frequency information within the wavelet domain contains the overall appearance or general characteristics of the tumor. Conversely, the high‐frequency information corresponds to the tumor's detailed features and finer aspects. Consequently, the low‐frequency coefficients are averaged, and the high‐frequency coefficients containing detailed information are summed to highlight the features. Finally, an element‐wise add operation is performed on the initial modal features to output the final fused features. These fused features are merged into the encoder and up‐sampled to output the segmentation result.

(1) $$\begin{array}{ccc} F & = & \mathrm{IDWT}(f(\mathrm{DWT}\left(X_{1}\right),\mathrm{DWT}\left(X_{2}\right),\\ & & \mathrm{DWT}\left(X_{3}\right),\mathrm{DWT}\left(X_{4}\right))) \end{array}$$

(2) $$X_{i}^{\prime }=X_{i}+F,i\in \left\{1,2,3,4\right\}$$

(3) $$\mathrm{Output}=\mathrm{Concat}(X_{1}^{\prime },X_{2}^{\prime },X_{3}^{\prime },X_{4}^{\prime })$$

where $X_{i}$ is the feature (i∈{1,2,3,4}) from the multi‐encoder, Low and high‐frequency subbands are acquired using the DWT, and fusion features F are created by fusing the subbands according to a fusion rule f. The initial features $X_{i}$ are added to these fusion features to create multimodal fusion feature $X_{i}$ for each i∈{1,2,3,4}. Ultimately, the output is obtained by concatenating these four modal features.

### 3.3 3D global context‐aware module

**FIGURE 5:**
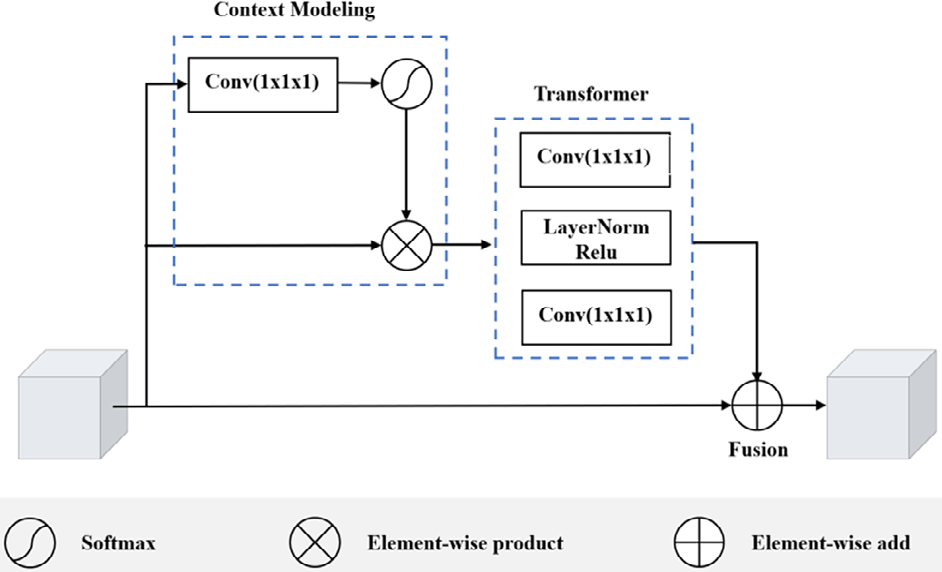
The 3D global context‐aware module.

We apply global context‐aware block (GCAM) extension to 3D images as shown in Figure 5. GCAM performs global context modeling on feature S. It mainly includes three processes: (a) context modeling; (b) transform; (c) fusion. As shown in the following equation.

(4) $$L_{\mathrm{context}}=S⊗\mathrm{Sotfmax}\left(\mathrm{Con}v_{1x1x1}\left(S\right)\right)$$

(5) $$\begin{array}{ccc} & & L_{\mathrm{trans}}\\ & & =\mathrm{Con}v_{1x1x1}\left(Re\mathrm{lu}\left(\mathrm{LayerNorm}\left(\mathrm{Con}v_{1x1x1}\left(L_{\mathrm{context}}\right)\right)\right)\right)\; \end{array}$$

(6) $$L_{\mathrm{fusion}}=S+L_{\mathrm{trans}}$$

where $L_{\mathrm{context}}$ is global attention pooling for context modeling, and ⊗ is element‐wise product. $L_{\mathrm{trans}}$ is to capture channel‐wise dependencies. The final output $L_{\mathrm{fusion}}$ is the sum of S and $L_{\mathrm{trans}}$.

### 3.4 Loss function

To alleviate the category imbalance problem,^40^ this article implements a strategy that merges the Binary CrossEntropy (BCE) Loss and the Dice loss. The complete loss function is formulated as follows.

(7) $$L_{\mathrm{BCE}}\left(u,v\right)=-\left(ulog\left(v\right)+\left(1-u\right)log\left(1-v\right)\right)$$

(8) $$L_{\mathrm{Dice}}\left(u,v\right)=1-\frac{2|u\cap v|+\varepsilon }{|u|+|v|+\varepsilon }$$

(9) $$L_{\mathrm{Total}}=L_{\mathrm{BCE}}+L_{\mathrm{Dice}}$$

where $L_{\mathrm{BCE}}$ and $L_{\mathrm{Dice}}$ represent the BCE loss and Dice loss, respectively. The values u and v denote the image label's pixel values and the predicted value for the target, respectively. ε is a small value incorporated to prevent division by zero.

## 4 EXPERIMENT

### 4.1 Dataset

We conducted experiments utilizing the publicly accessible brain glioma datasets of BraTS2018 and BraTS2021.^41^ The Brats2018 dataset consists of 285 samples in the training set and 66 samples in the validation set. The Brats2021 dataset comprises 1251 samples in the training set and 219 in the validation set. Each sample contains four modalities (T1, T1c, T2, and FLAIR) and measures 240×240×155. The training set samples have been expert annotated^42^ and are divided into three regions: enhanced tumor (ET), WT, and TC. The validation set does not contain true values, and researchers are required to submit their segmentation results for evaluation on the CBICA online platform.

### 4.2 Evaluation metrics

In validating our model, we employed established assessment metrics, including the Dice similarity coefficient and Hausdorff95. The Dice similarity coefficient quantifies the extent of concurrence between the predicted segmented area and the ground truth region. Meanwhile, the Hausdorff95 computes distances by considering the 95th percentile, gauging shape similarity by assessing distances between the predicted and actual ground truth regions. The mathematical formulations for these evaluation metrics are outlined as follows.

(10) $$\mathrm{Dice}=\frac{2\mathrm{TP}}{2\mathrm{TP}+\mathrm{FP}+\mathrm{FN}}$$

The variables TP, FN, and FP, respectively, represent the number of voxels that are true positive, false negative, and false positive.

(11) Hausdorff=max{h(P,Q),h(Q,P)}

P denotes the pixel set of prediction pixels, and Q represents the pixel set of ground truth. For P and Q, which can be expressed as $\left\{p_{1},p_{2},\text{…},p_{n}\right\}$ and $\left\{q_{1},q_{2},\text{…},q_{n}\right\}$, both in Euclidean space, the $h\left(P,Q\right)=\max_{p\in P}\min_{q\in Q}||p-q||$.

### 4.3 Implement details

The proposed model has been implemented using PyTorch. Due to GPU memory limitations, sample sizes were randomly cropped from (240, 240, 155) to (128, 128, 128). Additionally, the samples underwent random mirror flips with a 0.5 probability across axial, coronal, and sagittal planes, along with intensity shifts at a factor of 0.1. Starting at a learning rate of 1e‐5, the network was trained for 100 epochs using the Adam optimizer. As the epochs grew, the polynomial decay approach was followed by the learning rate decay. The batch size was fixed at 1, while the weight decay was set to 1e‐4. Ultimately, the z‐score standardization method^43^ was utilized to normalize the intensity values to a standardized scale, defined as follows:

(12) $$Z=\frac{m-\mu }{\sigma }$$

where m, μ, and σ represent the intensity, average, and standard deviation values of each pixel, respectively.

We separated the BraTS2018 and BraTS2021 training sets in a ratio of 4:1, with one part serving as a validation set for tuning the parameters and monitoring the model performance. The remaining sections serve as the training set for training the network parameters. For post‐processing, we adopted the same protocol as Brats2021 Champions,^44^ which involves converting ET regions smaller than 200 voxels to necrotic tumors to improve the overall dice coefficient. Additionally, to substantiate the efficacy of the models, we submit the predictive outcomes of the validation sets to the official platform for comprehensive evaluation.

### 4.4 Ablation experiments

**FIGURE 6:**
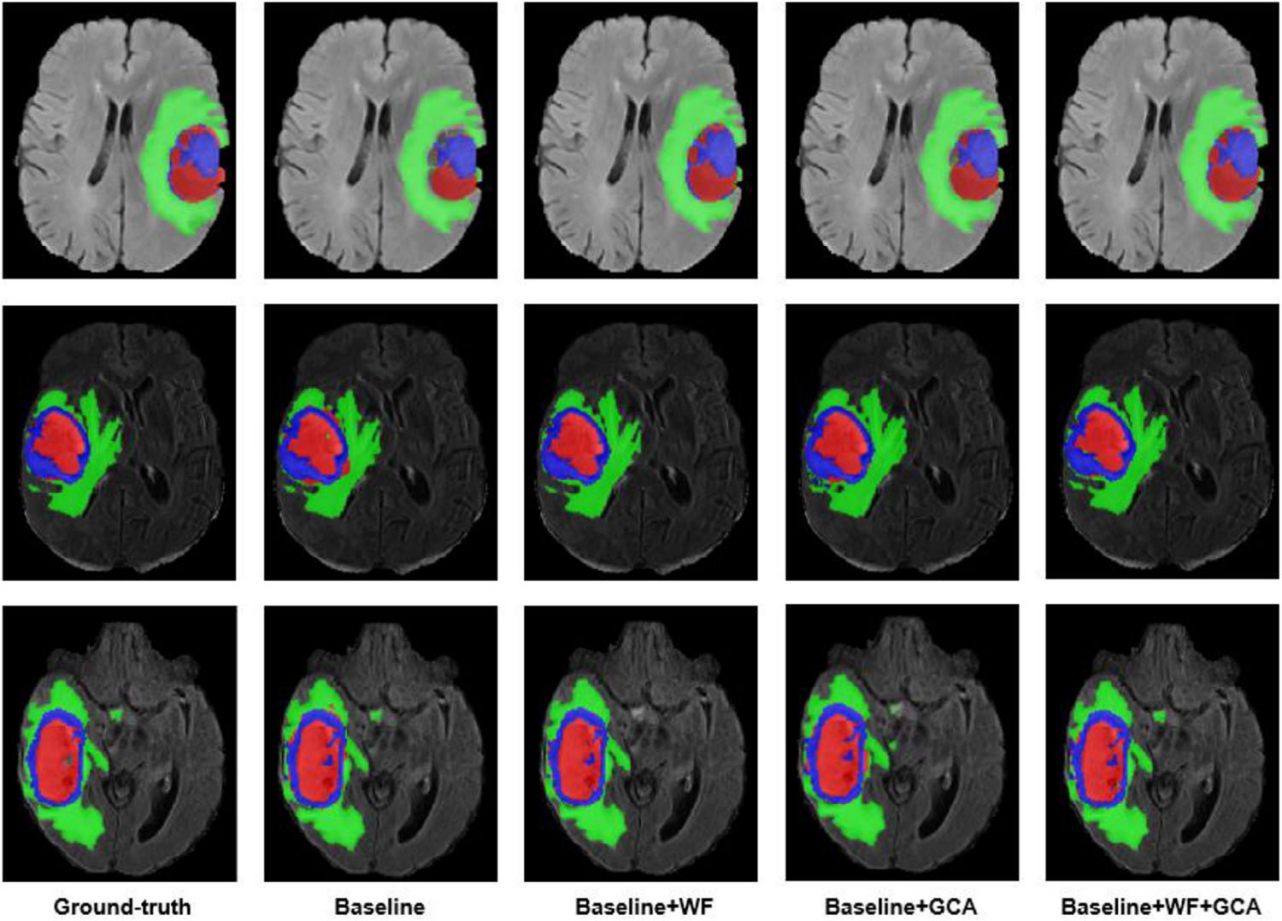
Visual segmentation results of ablation experiments. The non‐enhancing area is red, green indicates edema, and blue represents the enhancing tumor.

We validated our approach on the BraTS2021 dataset using a 3D Muti‐Encoder CNN as the baseline model. As depicted in Table 2, the integration of the WFM (WAM) into the baseline model resulted in an increase in the Dice similarity coefficients for ET, WT, and TC by 2.7%, 1.9%, and 6.1%, respectively. Simultaneously, the Hausdorff95 metrics were reduced by 1.69 , 2.66 , and 4.76 mm, corresponding to ET, WT, and TC, respectively. Subsequently, by incorporating the GCAM into the architecture above, there was an improvement observed in the Dice coefficients for ET and WT by 1.2% and 0.3%, respectively. Moreover, the Hausdorff95 is reduced by 0.67 , 0.98 , and 0.04 mm for each region. This effectively demonstrates the ability of the proposed components to enhance performance. Figure 6 visually illustrates that the proposed model produces segmentation results closely resembling the ground truth, particularly in terms of finer details.

**TABLE 2:** Quantitative results of ablation experiments on BraTS2021 training dataset, with the best results in **bold**.

	Dice (%)↑			Hausdorff95 (mm)↓		
Method	ET	WT	TC	ET	WT	TC
Baseline	0.812	0.900	0.827	13.45	8.75	11.96
Baseline + WFM	0.839	0.919	**0.888**	11.76	6.09	7.20
Baseline + GCAM	0.846	0.909	0.858	11.12	5.96	8.09
Baseline + WFM + GCAM	**0.851**	**0.922**	0.880	**11.09**	**5.11**	**7.16**

### 4.5 Experiment result

**FIGURE 7:**
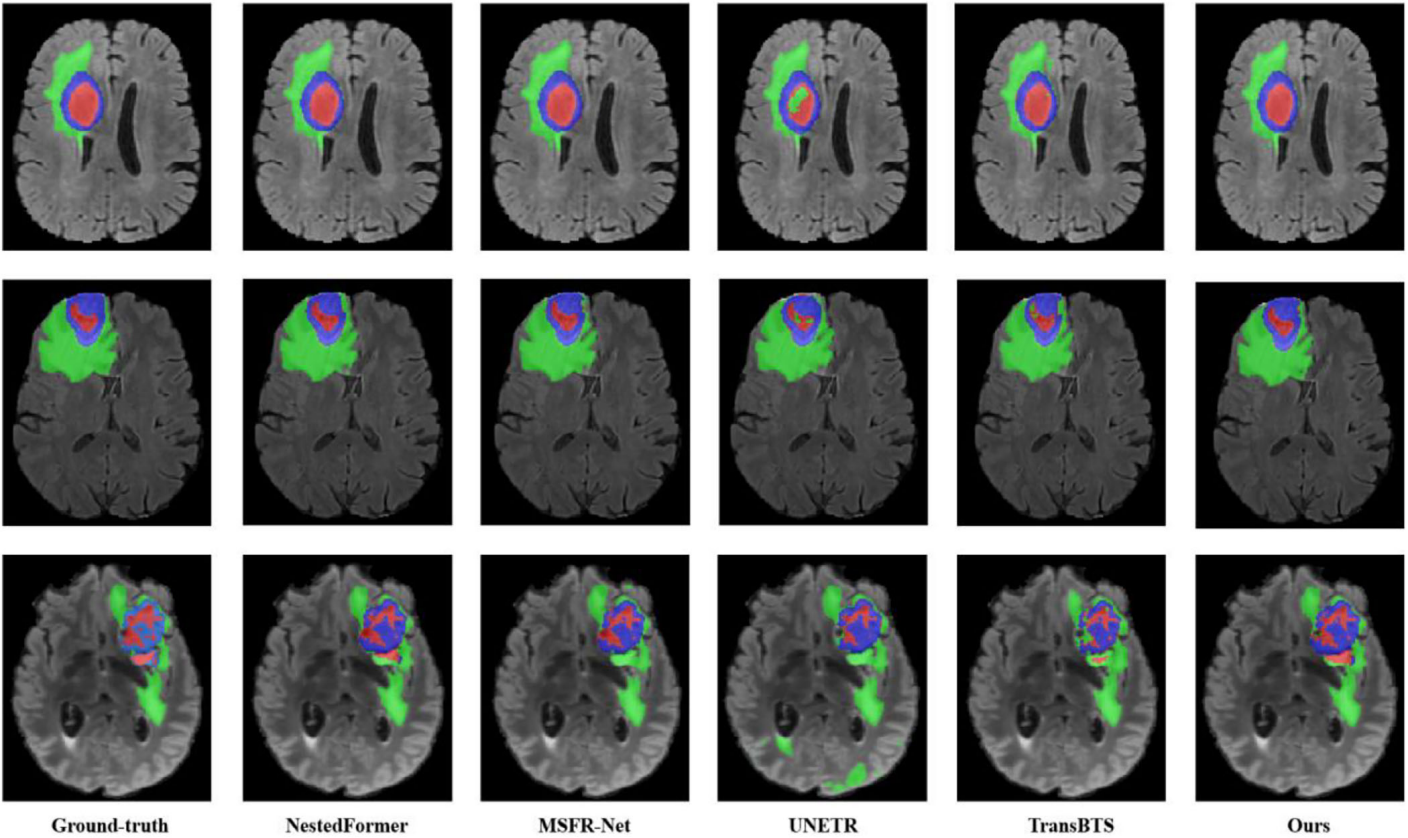
The segmentation results for several approaches are visualized. Red: non‐enhancing area, Green: edema, Blue: enhancing tumor.

To verify the superiority of our proposed method, we reproduce some classical models on the BraTS2018 and BraTS2021 datasets, which are briefly described in Table 3. Our proposed method employs a late fusion strategy, which can fully consider the complementary information of the multimodalities compared to the early fusion. It also utilizes the wavelet transform module to combine spatial and wavelet domain features for simple and efficient fusion without designing complex multimodal fusion networks as in NestedFormer and MSFR‐Net. Meanwhile, leveraging the self‐attention mechanism, which accounts for both the entire tumor region and sub‐region segmentation, our method achieves superior performance in the WT and ET regions in terms of the Dice coefficient. Although the performance in the TC region is slightly lower than that of other methods, it remains competitive, as demonstrated in Table 4. This can also be reflected in Figure 7, the first two rows represent simple segmentation examples. In all cases, the segmentation results are similar to the ground truth, except for UNETR, which is poor. In the third row, where the tumor segmentation is more complex, the performance of NestedFormer is comparable to our method, while the remaining models perform relatively unsatisfactory.

**TABLE 3:** A brief description of the methods used for performance comparison in our experiments.

Method	Fusion strategy	Brief description
NestedFormer^24^	Later fusion	Designing complex nested network structures concerned with modal feature fusion.
MSFR‐Net^25^	Later fusion	Designing independent convolutional networks for Flair and T1 for modal fusion and failing to capture the long‐range dependence of tumors. tumor long‐range dependencies.
UNETR^15^	Early fusion	Use the Transformer directly as an encoder with high computational overhead for training and inference.
TransBTS^16^	Early fusion	Embedding Transformer encoder in the last layer of a U‐shaped convolutional network to balance global and local features.
Ours	Later fusion	Combine spatial and wavelet domain features to fuse modalities and capture complementary information between modalities.

**TABLE 4:** Comparison results on the BraTS2018, BraTS2021 training dataset. The evaluation metrics are the dice score and Hausdorff distance for each sub‐region of the prediction results, with the best results in **bold**.

		Dice (%)↑			Hausdorff95 (mm)↓		
Dataset	Method	ET	WT	TC	ET	WT	TC
BraTS2018	NestedFormer^24^	0.746	0.907	0.765	**24.69**	4.50	**7.63**
	MSFR‐Net^25^	0.721	0.906	**0.796**	45.48	6.09	10.20
	UNETR^15^	0.660	0.828	0.645	47.10	11.83	20.64
	TransBTS^16^	0.699	0.832	0.709	42.06	12.98	21.82
	Ours	**0.776**	**0.910**	0.782	29.87	**4.37**	13.68
BraTS2021	NestedFormer^24^	0.846	**0.922**	**0.885**	12.00	5.32	7.21
	MSFR‐Net^25^	0.850	0.915	0.882	**10.15**	6.47	7.19
	UNETR^15^	0.824	0.899	0.837	11.74	7.95	11.92
	TransBTS^16^	0.827	0.895	0.850	11.21	7.87	8.35
	Ours	**0.851**	**0.922**	0.880	11.09	**5.11**	**7.16**

**FIGURE 8:**
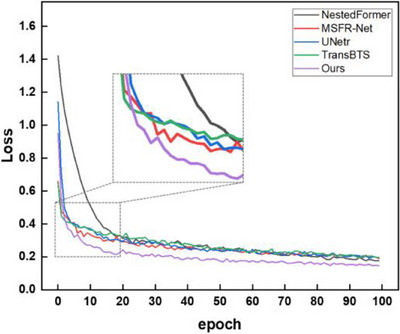
Comparison of the convergence speed of different model loss curves at 100 epochs.

**FIGURE 9:**
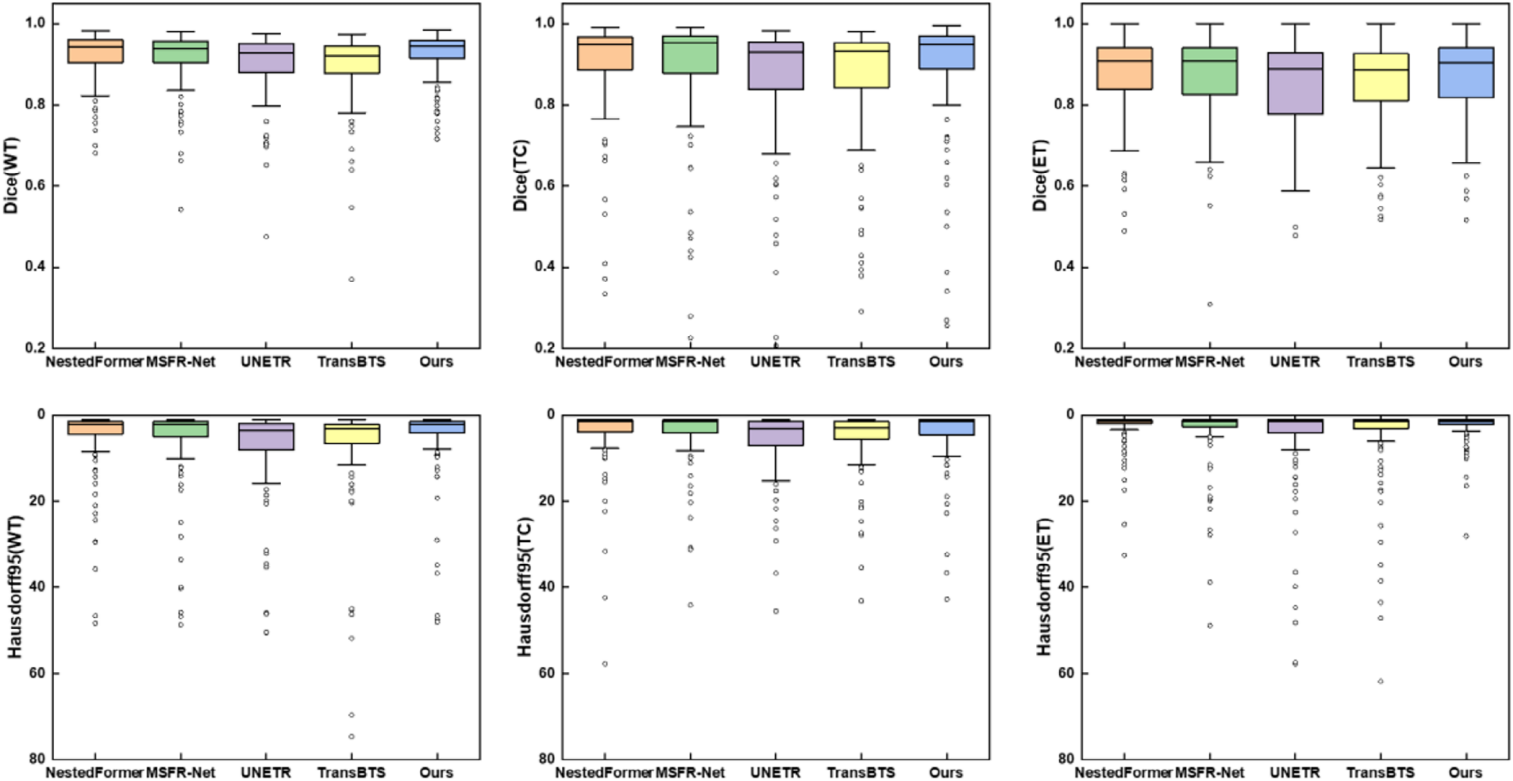
Boxplots are utilized to facilitate the comparison of various methods concerning both Dice metrics and Hausdorff95 Distance metrics. Dots show outliers.

A comparative investigation was conducted, presenting the computation and parameter analysis for an input image size of 128 × 128 × 128, as detailed in Table 5. Our model generally establishes a balance between the quantity of parameters and the efficacy of segmentation. Figure 8 illustrates the relatively swifter convergence of our model's loss function on the BraTS2021. The boxplots depicted in Figure 9 allow for a comparison across various methods regarding both Dice and Hausdorff Distance metrics. Interestingly, our suggested approach shows fewer outliers than the other methods, indicating a strong learning ability and improved robustness to complex samples in our model.

**TABLE 5:** Results were compared with the models mentioned above for flops and parameters when the array of 128 × 128 × 128 was input.

Methods	GFlops	Param.
NestedFormer	209.27	10.29
MSFR‐Net	739.24	15.01
UNETR	2176.88	81.84
TransBTS	168.45	7.36
Ours	440.50	10.39

**FIGURE 10:**
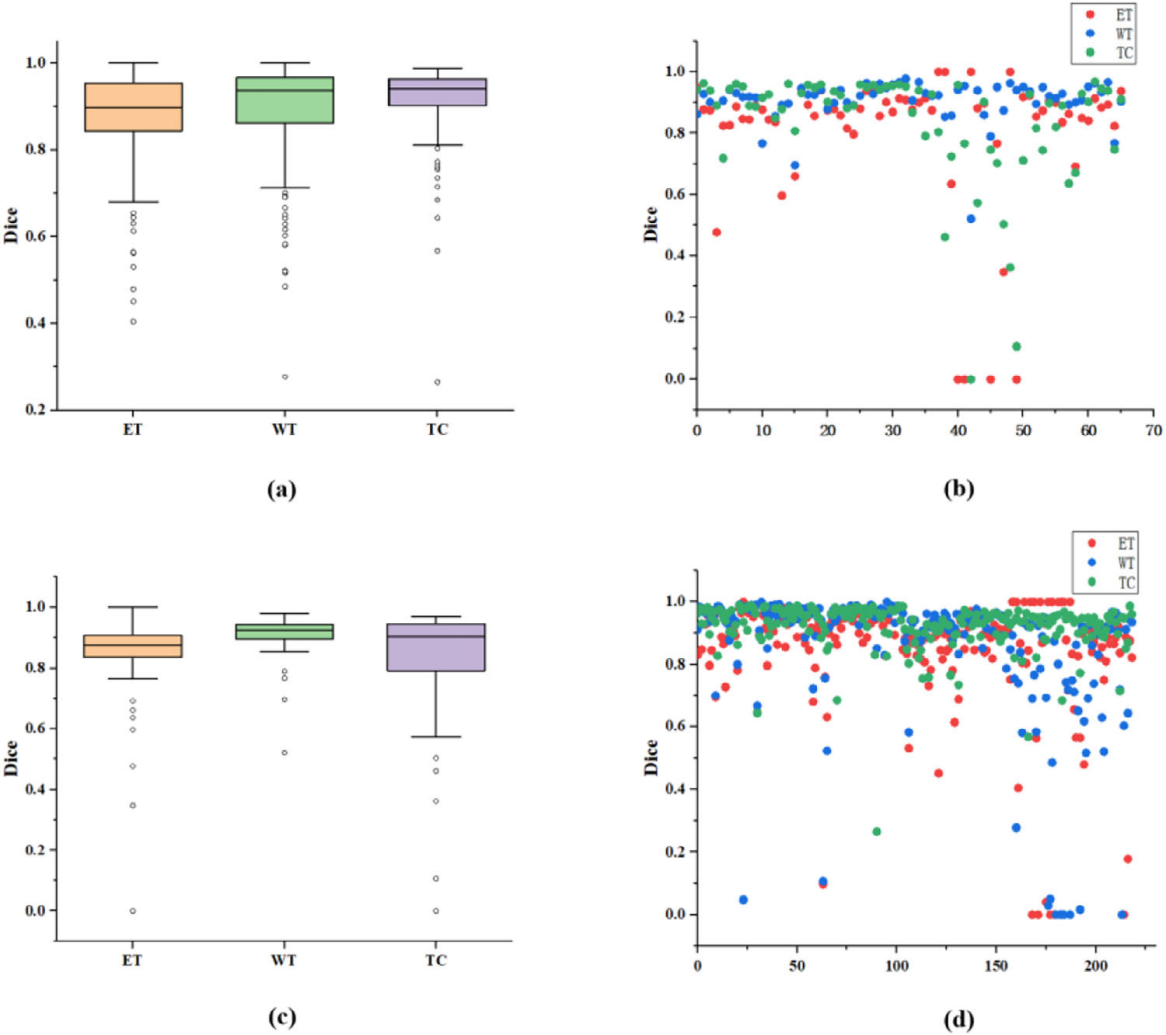
Boxplot and scatter plots of Dice coefficients. (a) and (b) are from the BraTS2018 online validation set, and (c) and (d) are from the BraTS2021 online validation set.

From Table 6, we further validate our model by comparing it to the latest models that employ an early fusion strategy that focuses on designing complex network structures to enhance segmentation. Our approach focuses on the intermodal fusion process, combining wavelet‐domain and spatial‐domain features, and achieves a clearer advantage in several segmentation metrics. On the online BraTS2018 validation dataset, the dice coefficient of the proposed method is optimal in the ET and WT regions, and the Hausdorff distance is optimal in three ET, WT, and TC regions. In contrast, the DPAFNet^17^ employs a two‐path model and a multiscale attention fusion strategy for tumor segmentation, resulting in a dice coefficient that is 0.6% more than our method. On the online BraTS2021 validation dataset, our dice coefficient and Hausdorff distance are more dominant in the ET region and slightly less dominant in the other regions. Some methods are dominant in a few metrics, for example, E1D3 U‐Net^45^ tackles brain tumor subregion segmentation by using a multi‐decoder architecture to decode the ET, WT, and TC regions of the tumor individually, reaching 9.61 in the TC region at the Hausdorff distance, and 3D PSwinBTS^46^ achieves better segmentation by combining the inductive bias property of convolution with the self‐attention‐based long‐range capturing capability to achieve better segmentation results. Compared with the above methods, our method has notable performance in terms of average Dice and average Hausdorff distance in each tumor region. Figure 10 displays the boxplot and scatter plots representing the Dice coefficients obtained from the submission to the BraTS2018 and BraTS2021 online validation sets.

**TABLE 6:** Comparative performance on BraTS2018, BraTS2021 online official validation dataset, with the best results in **bold**.

		Dice (%)↑				Hausdorff95 (mm)↓			
Dataset	Method	ET	WT	TC	Avg	ET	WT	TC	Avg
BraTS2018	Cascade V‐net^47^	0.777	0.905	0.836	0.839	3.51	5.18	6.28	4.99
	CANet^48^	0.767	0.898	0.834	0.833	3.86	6.69	7.67	6.07
	Sun et al.^49^	0.772	0.892	0.763	0.809	5.04	5.48	10.62	7.05
	DPAFNet^17^	0.795	0.900	**0.839**	0.844	2.92	6.51	5.71	5.05
	Ours	**0.804**	**0.906**	0.833	**0.848**	**2.73**	**4.52**	**5.38**	**4.21**
BraTS2021	Akbar et al.^50^	0.777	0.893	0.821	0.830	30.90	11.34	16.09	19.44
	3D ResUNet^51^	0.819	0.899	0.850	0.856	17.89	4.30	9.89	10.69
	E1D3 U‐Net^45^	0.822	0.924	0.865	0.870	19.73	4.23	**9.61**	11.19
	BiTr‐Unet^52^	0.818	0.909	0.843	0.857	17.84	4.50	16.68	13.01
	3D PSwinBTS^46^	0.826	**0.926**	**0.867**	0.873	17.53	**3.73**	11.08	**10.78**
	Ours	**0.846**	0.921	0.854	**0.874**	**14.48**	4.76	15.00	11.41

## 5 CONCLUSION

This article introduces an automatic segmentation model for MRI multimodal brain tumors. This model includes a multi‐encoder single‐decoder structure that uses a 3D DWT to extract multimodal features for fusion. Additionally, we have included a global context‐aware module that captures global information and integrates low‐level semantic data with high‐level semantic features. The ablation experiment conducted effectively showcases the efficacy of the proposed module. Additionally, through comparison experiments performed on the BraTS2018 and BraTS2021 validation sets, our findings exhibit competitiveness when evaluated against prevalent modeling approaches.

## AUTHOR CONTRIBUTIONS

Yuheng Pan and Weijia Lu are involved in writing, reviewing, and editing the original draft and supervision. Haohan Yong implemented the deep learning segmentation models and is involved in drafting the manuscript. The review and editing of the draft were done by Guoyan Li and Jia Cong. The authors contributed equally to the preparation of the manuscript and the concept of the research.

This study was funded by the National Natural Science Foundation of China(No. 62204168, the Tianjin Science and Technology Research Project (No. 20YDTPJC00160, No. 21YDTPJC00780), the Science Research Program of Tianjin Education Committee (No. 2019KJ101), and Tianjin Research Innovation Project for Postgraduate Students (No. 2022KYZ136).

## CONFLICT OF INTEREST STATEMENT

The authors declare no conflicts of interest.
